# The burden of hepatitis C virus in the world, China, India, and the United States from 1990 to 2019

**DOI:** 10.3389/fpubh.2023.1041201

**Published:** 2023-03-02

**Authors:** Jia Yang, Jin-Lei Qi, Xiao-Xiao Wang, Xiao-He Li, Rui Jin, Bai-Yi Liu, Hui-Xin Liu, Hui-Ying Rao

**Affiliations:** ^1^Beijing Key Laboratory of Hepatitis C and Immunotherapy for Liver Diseases, Peking University Hepatology Institute, Peking University People's Hospital, Beijing, China; ^2^National Center for Chronic and Non-communicable Disease Control and Prevention, Chinese Center for Disease Control and Prevention, Beijing, China; ^3^Department of Clinical Epidemiology and Biostatistics, Peking University People's Hospital, Beijing, China

**Keywords:** incidence, mortality, DALYs, global burden disease, hepatitis C

## Abstract

**Background and aim:**

Hepatitis C virus infection can lead to an enormous health burden worldwide. Investigating the changes in HCV-related burden between different countries could provide inferences for disease management. Hence, we aim to explore the temporal tendency of the disease burden associated with HCV infection in China, India, the United States, and the world.

**Methods:**

Detailed data on the total burden of disease related to HCV infection were collected from the Global Burden of Disease (GBD) 2019 database. Joinpoint regression models were used to simulate the optimal joinpoints of annual percent changes (APCs). Further analysis of the age composition of each index over time and the relationship between ASRs and the socio-demographic Index (SDI) were explored. Finally, three factors (population growth, population aging, and age-specific changes) were deconstructed for the changes in the number of incidences, deaths, and DALYs.

**Results:**

It was estimated that 6.2 million new HCV infections, 0.54 million HCV-related deaths, and 15.3 million DALYs worldwide in 2019, with an increase of 25.4, 59.1, and 43.6%, respectively, from 1990, are mainly due to population growth and aging. China experienced a sharp drop in age-standardized rates in 2019, the United States showed an upward trend, and India exhibited a fluctuating tendency in the burden of disease. The incidence was increasing in all locations recently.

**Conclusion:**

HCV remains a global health concern despite tremendous progress being made. The disease burden in China improved significantly, while the burden in the United States was deteriorating, with new infections increasing recently, suggesting more targeted interventions to be established to realize the 2030 elimination goals.

## 1. Introduction

Hepatitis C virus (HCV) could result in acute and chronic hepatitis, leading to injuries ranging from minor ailments to severe, stubborn diseases involving hepatic cirrhosis and carcinoma. Because of the insidious nature of HCV infection, only 20% of patients infected with HCV were diagnosed and only 15% of those diagnosed received treatment (1). Thus, HCV infection remains one of the leading causes of liver diseases worldwide. According to the WHO, there are 58 million HCV infections worldwide, with approximately 1.5 million new HCV infections annually and 290,000 deaths resulting from HCV infection (2). Although there is no available protective vaccine at present (3), direct-acting antivirals (DAAs) have significantly revolutionized the treatment of HCV infection, making treatment the primary means of management of the infectious disease (4–7). In this context, the World Health Assembly proposed to eliminate viral hepatitis as a public health threat by 2030 in 2016 (8). However, due to the impact of the COVID-19 epidemic and other factors, most countries failed to meet their expected goals by 2020 (9). Thus, the WHO developed interim guidance with absolute incidence targets (new HCV infections in 100,000 persons per year) in 2021 (10). There are still many obstacles to realizing this strategic goal, lacking relevant epidemiological data being an important factor. Many countries and regions lack a complete disease surveillance system and epidemiological data related to HCV infection, so it is difficult to formulate targeted strategies (8, 11).

At the national level, improving the disease surveillance system, assessing the national HCV disease burden, and providing accessible medical services to all regions and populations are three important directions of action (8, 12, 13). However, data regarding the burden of HCV among specific nations are lacking, and most previous studies were mainly based on regional comparative analysis (14–16). Nevertheless, disease management is mainly based on the country as a unit, and situations among nations vary (14, 15, 17). In addition, there are also common dilemmas in different countries and experiences that can be learned from each other. China, India, and the United States are the three most populous countries around the world, accounting for more than 40% population worldwide. They are also among the top three countries for the burden of disease associated with HCV infection (Egypt dropped out of the top three in 2019) (18, 19). China and India share many things in common: Both are developing countries and located in Asia with a heavy burden of HCV infection (15, 20, 21). At the same time, HCV is the major cause of chronic liver diseases in the United States, and it seems that there is a trend of further deterioration (22, 23). In addition, the United States is a representative of developed countries, and its current situation may be the future of developing countries. Comparing the HCV burden among these countries could provide valuable information for the elimination of HCV infection and useful references for other counties.

Therefore, this study aims to explore the changing trend of morbidity, mortality, and DALYs of HCV infection-related disease burden in China, India, the United States, and the world from 1990 to 2019, to draw experience and lessons for policymakers and provide the basis for developing targeted measures to accelerate global progress toward 2030 goals.

## 2. Materials and methods

### 2.1. Overview

The Global Burden of Disease (GBD) 2019 estimated all epidemiological indicators of 369 diseases and injuries in 204 countries and regions around the world from 1990 to 2019, including incidence, prevalence, mortality, years lived with disability (YLDs), years of life lost (YLLs), and disability-adjusted life years (DALYs) in different sexes and age groups, as well as corresponding age-standardized rates. Each indicator was estimated by modeling with standardized tools: Cause of Death Ensemble model (CODEm), spatiotemporal Gaussian process regression (ST-GPR), and DisMod-MR15 are shown with 95% uncertainty intervals (UIs). Detailed GBD data estimation models and methods can be found in previously published literature (24–26).

### 2.2. Data resources

Data on the number and age-standardized rate of incidence, prevalence, death, YLL, YLD, and disability-adjusted life years (DALYs) of “Total burden related to hepatitis C” in the world and three countries (China, India, and United States) were extracted from GBD 2019 (http://ghdx.healthdata.org/gbd-2019), as well as the corresponding population numbers at 5-year intervals. The International Classification of Diseases Ninth Revision (ICD-9) and ICD Tenth Revision (ICD-10) codes defining “Total burden related to hepatitis C” are listed in Supplementary Table S1. This database is a public database and does not require ethics approval.

### 2.3. Socio-demographic Index

The socio-demographic Index (SDI) was generated by the geometric mean of rescaled values of the total fertility rate of women under the age of 25, the average educational year of the population aged 15 and older, and lag-distributed income per capita, representing the socioeconomic development level of the region (27). The values of SDI range from 0 (worst) to 1 (best). 0 represents the lowest level of socioeconomic development, while 1 represents the highest level of socioeconomic development. We explored the association between HCV-related age-standardized rates (ASRs) and SDI. Data for SDI were extracted from the GBD 2019 (Supplementary Tables S3, S4).

### 2.4. Data analysis

#### 2.4.1. Temporal trends in ASRs

Annual percent change (APC), average APC (AAPC), and the corresponding 95% CI between 1990 and 2019 in HCV-related age-standardized rates were calculated by the Joinpoint regression program, version 4.9.1.0, using 5-year age groups including those younger than 1 year old and those over 95 years old. The 95% UI was divided by 1.96 × 2 to obtain the corresponding standard errors. The Joinpoint model will select the turning points of the best-fit model according to the Monte Carlo Permutation Method, and the maximum turning point is set to 2. APC is statistically significant compared to 0, which means that ASRs have changed over time during this period (or increased or decreased), and no statistical significance means that ASRs have not changed over time during this period (14, 28).

#### 2.4.2. Decomposition of changes

The changes in the number of incidences, deaths, and DALYs from 1990 to 2019 in each region were decomposed into three factors—population growth, population aging, and age-specific change (29). The net overall change, that is, the absolute change in the number of incidences, deaths, and DALYs, is also equal to the arithmetic sum of the changes in the three factors. First, assume two unrealistic scenarios. The first scenario assumes that the population has grown from 1990 to 2019, but the other two factors, population aging and age-specific changes, remain unchanged over time. From this, the hypothetical 2019 population with only the participation of population growth factors can be obtained. The second scenario multiplies the age-specific rate in 1990 by the actual age-related population structure in 2019 to obtain the assumed population in 2019 (only population growth and aging factors are involved). The population difference between the two scenarios reflects changes in the burden of disease caused by population aging exclusively. The difference between the first scenario and the actual population in 1990 reflects changes due to population growth, while the difference between the actual value in 2019 and the second scenario reflects changes attributable to age-specific rates (30, 31).

R software, version 4.1.0 was used for data analysis and graphing, and Joinpoint regression software, version 4.9.1.0 was used to obtain the time trend of each indicator.

## 3. Results

### 3.1. Burden of HCV infection in China, India, the United States, and the world, 2019

Globally, in 2019, there were estimated to be 6.2 million new HCV infections, 540,000 HCV infection-related deaths, and 15.3 million HCV infection-related DALYs, with an increase of 25.4, 59.1, and 43.6%, respectively, compared to 1990. The number of global new HCV infections in 2019 is similar to that of men and women, but the deaths and DALYs of men are higher than those of women, and the increase is also greater. New infections in India accounted for nearly one-sixth of new infections worldwide, followed by China. Meanwhile, India had the highest DALYs associated with HCV infection among the three countries, while China had the greatest number of HCV infection-related deaths. Men had higher incidences, deaths, and DALYs than women in all three countries. The United States had the highest percentage increase in disease burden from 1990 to 2019 among the three countries, and US women had the largest increase. At the same time, the number of new HCV infections, related deaths, and DALYs in Chinese women decreased significantly with less decline in men (Table 1 and Supplementary Table S2).

**Table 1:** Incidence and disability-adjusted life year, age-standardized rates, and their temporal trends of HCV infection in different regions, 1990 vs. 2019.

**Country**	**Sex**		**Incidence**													**Disability-adjusted life year**								
			**Number in** **Thousands** **(95%UI)**				**Percentage** **change** **in number** **(95% UI)**		**Age-** **standardized** **rate** **per 100,000** **(95%UI)**			**Percentage** **change** **in rate** **(95% UI)**				**Number in** **thousands** **(95%UI)**			**Percentage** **change** **in number** **(95% UI)**		**Age-** **standardized** **rate** **per 100,000** **(95%UI)**		**Percentage** **change** **in rate** **(95% UI)**	
			**1990**	**2019**			**1990–2019**		**1990**	**2019**		**1990–2019**				**1990**	**2019**		**1990–2019**		**1990**	**2019**	**1990–2019**	
Global	Overall		4959.9 (4414.8-5662.5)	6218.6 (5552.1-7128.6)			25.4% (20.4~31.2%)		90.9 (81.1-103.9)	82.5 (73.4-94.7)		−9.3% (−10.6~−7.8%)				10645.6 (9325.6-11986.9)	15288.5 (13297.5-17477.9)		43.6% (31%-57.7%)		242.8 (212.8-272.7)	184.5 (160.7-210.2)	−24% (−30.1~−16.8%)	
		Male		2495.3 (2222-2852.9)	3176.4 (2830.8-3627.8)			27.3% (22.3~33.2%)		91.3 (81.5-103.9)	84.2 (75.1-96.1)		−7.8% (−9.3~−6.3%)				6921.2 (5993.8-7867.4)	10328.2 (8914.9-11908.5)		49.2% (35%-65.1%)		323 (281.5-366)	255.8 (221.6-293.3)	−20.8% (−27.9~−12.3%)	
		Female		2464.5 (2184.3-2832.7)	3042.2 (2709.4-3491.4)			23.4% (18.1~29.7%)		90.7 (80.6-104.1)	80.9 (71.7-93.4)		−10.8% (−12.1~−9.2%)				3724.4 (3198.2-4363.9)	4960.3 (4286.8-5623.5)		33.2% (15%-53.2%)		165.5 (142.9-192.2)	116.1 (100.5-131.7)	−29.9% (−38.1~−20%)	
China	Overall		996.5 (872-1142.9)	625.3 (551-711.8)			−37.3% (−41.7~−32%)		87.9 (77-100.6)	55 (47.9-63.2)		−37.5% (−39.7~−35.5%)				2397.8 (2033.5-2794.1)	2035.7 (1675.4-2429.6)		−15.1% (−32.1~6.2%)		253.8 (216.4-294.3)	100.8 (83.3-119.1)	−60.3% (−67.8~−50.7%)	
		Male		501.4 (438.5-574)	334.6 (293.2-380.4)			−33.3% (−38.2~−27.5%)		85 (74.8-97.1)	55.9 (48.8-64)		−34.2% (−36.6~−31.8%)				1494.7 (1199.1-1821.3)	1405.6 (1095.4-1755)		−6% (−29.7~28.4%)		304.2 (246.8-366.7)	142.4 (112.1-176.8)	−53.2% (−64.7~−37%)	
		Female		495 (431.5-565.7)	290.7 (256.3-333.7)			−41.3% (−45.5~−36.4%)		91.1 (79.7-104.1)	54.6 (47-63.2)		−40.1% (−42.6~−37.9%)				903.1 (732.3-1109.1)	630.1 (493-782.1)		−30.2% (−47.6~−6%)		199.2 (162.3-244.4)	60.3 (47.3-74.9)	−69.7% (−77.3~−59.1%)	
India	Overall		466.5 (404.8-550.6)	650 (565.2-757.3)			39.4% (29.7~50%)		56.2 (48.9-65.7)	50.7 (44.2-58.6)		−9.8% (−12.2~−7.1%)				1298.5 (1048.4-1666)	2310.2 (1884.4-2782.1)		77.9% (40.6~123.5%)		201.8 (166.9-254.5)	176.1 (144-211.6)	−12.8% (−28.7~7.6%)	
		Male		251.8 (217.7-298.7)	357.7 (309.4-418.4)			42% (32.8~52.1%)		57.1 (49.6-67)	53.3 (46.5-61.7)		−6.6% (−9.3~−3.5%)				863.6 (707.3-1083.5)	1605 (1243.3-2003.2)		85.8% (43.2~139.5%)		256.6 (211.9-318.3)	240 (187.2-299)	−6.5% (−26.6~19.6%)	
		Female		214.7 (186-253.3)	292.3 (253.6-342.6)			36.2% (25.9~48.2%)		55.1 (47.9-64.5)	47.5 (41.4-55.1)		−13.8% (−16.3~−11.2%)				434.9 (296.1-687)	705.2 (533-926.1)		62.2% (4.4~133.8%)		141.3 (103.7-204.8)	110.7 (83.9-145.9)	−21.7% (−44.1~8.1%)	
The United States	Overall		145.4 (126-167.9)	230.1 (202.2-262.9)			58.2% (49~68.3%)		56.2 (49.2-64.8)	66.5 (59.2-75.9)		18.4% (14.9~22.8%)				491.4 (446.6-541.7)	1045.7 (948.4-1153.8)		112.8% (102~123.4%)		171.1 (154.6-188.9)	220.6 (201.5-242.6)	28.9% (23.5~34.2%)	
		Male		82 (71.2-95)	124.7 (109.9-142.4)			52.2% (42.6~62.6%)		65.4 (57.2-75.3)	74 (66.1-84.4)		13.2% (9.5~17.9%)				343.6 (312.3-375.8)	705.8 (636.8-780.1)		105.4% (93.5~118%)		256.7 (233.1-281.8)	309.7 (281.2-340.7)	20.7% (14.4~26.9%)	
		Female		63.4 (54.9-73.2)	105.3 (91.8-121)			66.1% (57.3~76.2%)		47.2 (41.2-54.6)	59.1 (52.2-67.5)		25.2% (21.1~29.8%)				147.8 (131.3-166)	339.9 (302.6-378.2)		130% (117.4~144.6%)		93.8(83-105.9)	137 (122.3-152.3)	46.1% (39.3~54.4%)	

**Figure 1:**
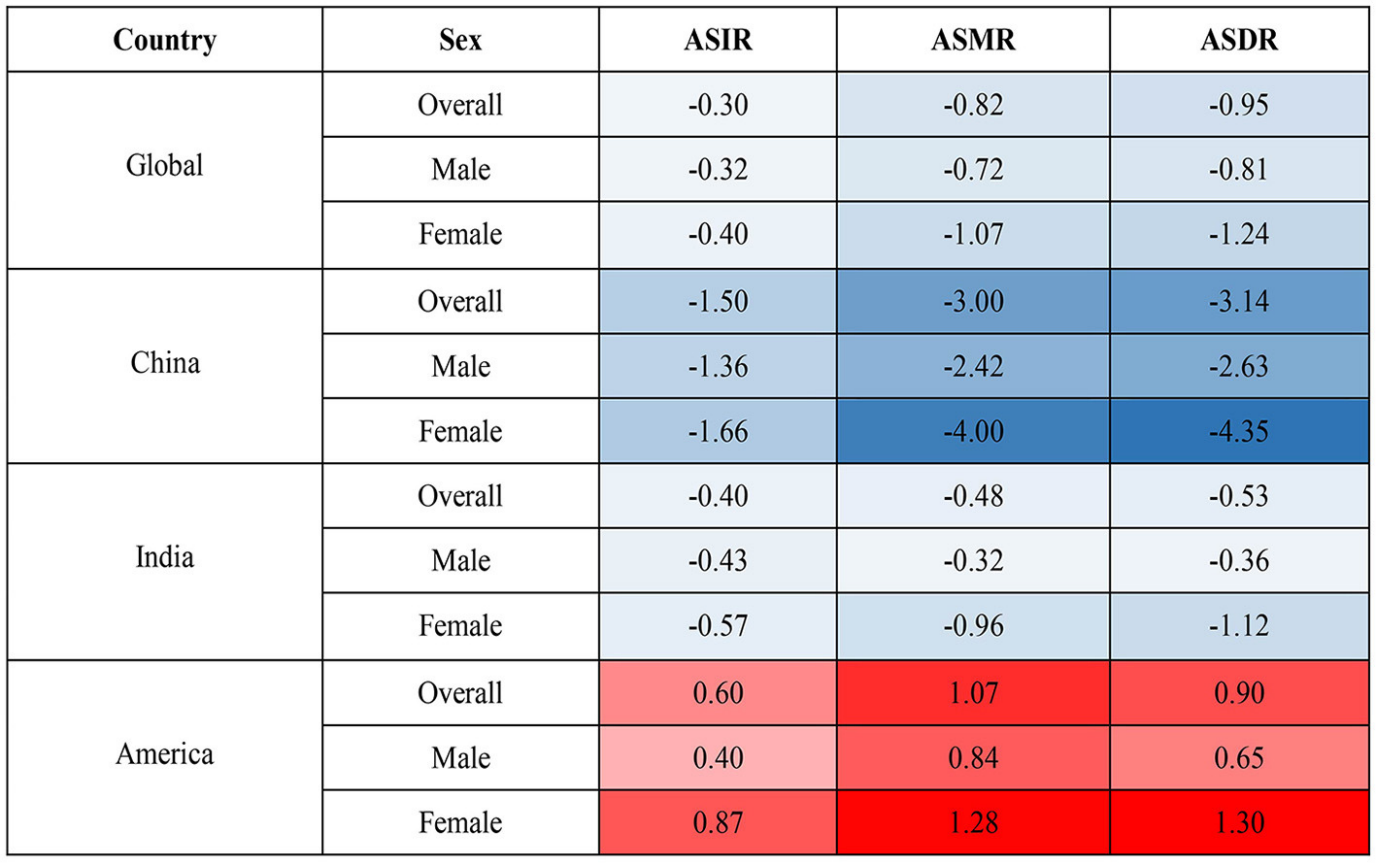
AAPC in ASIR, ASMR, and ASDR from 1990 to 2019. AAPC, average annual percent change; ASIR, age-standardized incidence rate; ASMR, age-standardized mortality rate; ASDR, age-standardized DALY rate.

On the contrary, the global age-standardized HCV infection incidence rate (ASIR, per 100,000), age-standardized mortality rate (ASMR, per 100,000), and age-standardized DALY rate (ASDR, per 100,000) in 2019 were 82.5 (95%UI 73.4–94.7), 6.7 (5.9–7.5), and 184.5 (160.7–210.2), and the average annual percent changes (AAPCs) of these three indicators from 1990 to 2019 were −0.30, −0.82 and −0.95, respectively. ASDR in India was similar to the global average, with the highest ASIR in the United States and the lowest in China. The ASMR and ASDR of the United States also ranked first among the three countries, with the highest increase ratio, and their corresponding AAPCs were 1.28 and 1.30, respectively. Meanwhile, the disease burden of HCV in China has dropped significantly, especially among women. Chinese women had the largest declines in ASDR and ASMR in HCV infection among the three countries, and the corresponding AAPCs were −4.00 and −4.35, respectively (Table 1, Supplementary Table S2, and Figure 1).

### 3.2. Changes in the disease burden of HCV infection from 1990 to 2019

**Figure 2:**
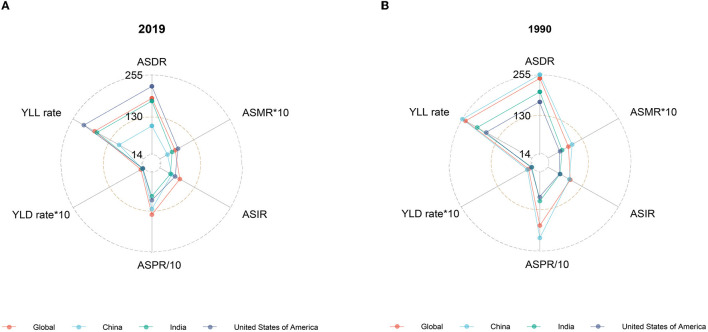
Burden of HCV in ASRs, 2019 vs. 1990. **(A)** Epidemiological indicators of China, India, the United States, and the world in 2019. **(B)** Epidemiological indicators of China, India, the United States, and the world in 1990.

The global disease burden of HCV infection has improved significantly since 1990. In 2019, all epidemiological indicators in the world decreased significantly compared with 1990. However, the trends are not consistent across countries. In 1990, China's ASMR, ASDR, age-standardized incidence rate (ASIR), years of life lost due to premature mortality (YLL) rate, and years lived with disability (YLD) rate ranked first among the three countries, slightly higher than the global ones, and the corresponding epidemiological indicators in the United States were the lowest, while India was between the United States and the world. In 2019, the ASRs in China decreased significantly, while the ASRs in the United States increased compared with 1990 except for ASPR and age-standardized YLD (Figure 2).

**Figure 3:**
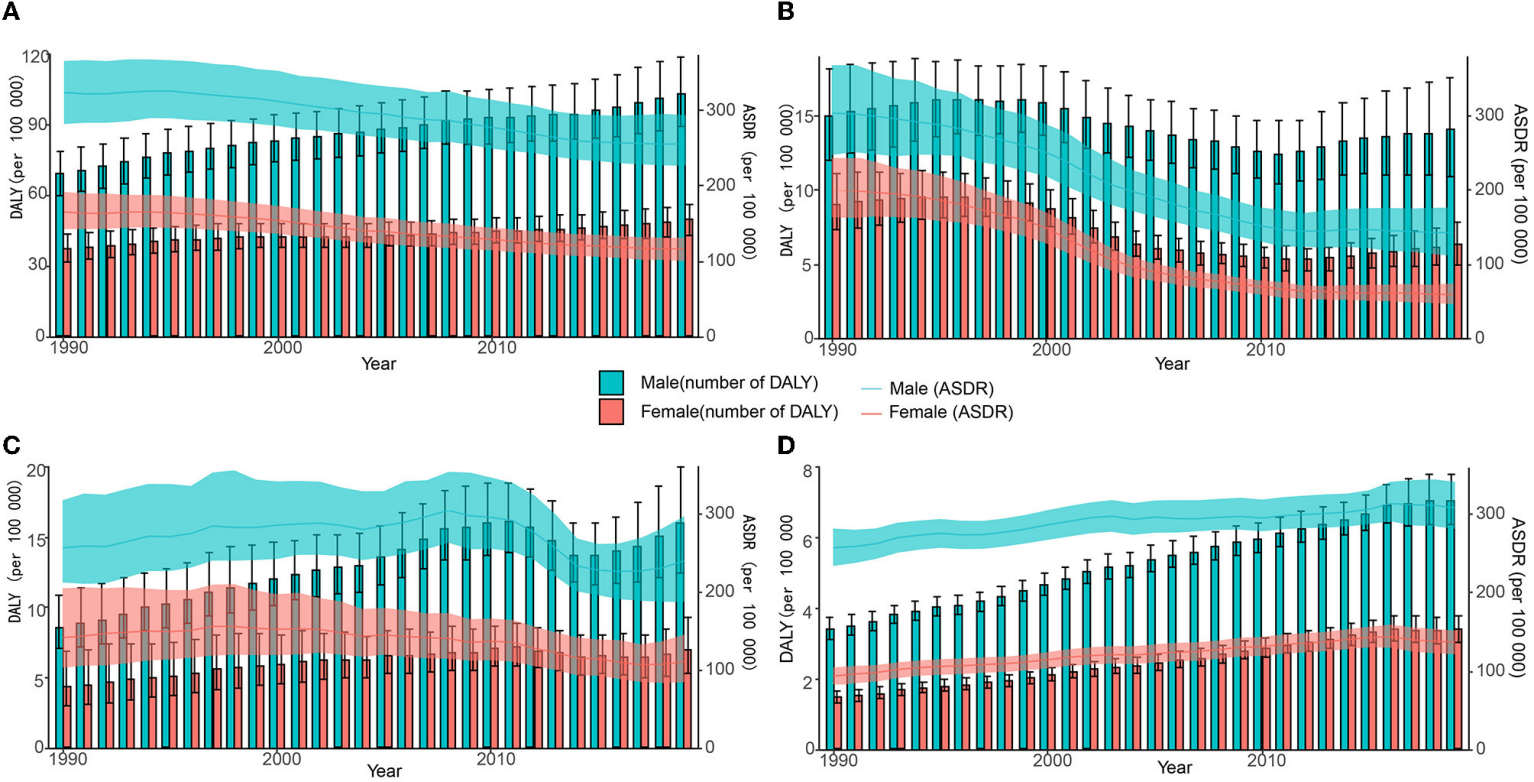
Number and age-standardized rate of DALY with 95% uncertainty intervals (UIs) in different regions, 1990–2019. **(A)** Global; **(B)** China; **(C)** India; **(D)** the United States.

Despite an increasing trend in absolute values of HCV infection-related DALYs worldwide, ASDR declined gradually, with the fastest decline occurring between 1997–2016 (men) and 1996–2013 (women). The changing trend of ASDR and DALY in China is similar to that of the world, but the ASDR declines faster, and the periods with the largest decline rates are 1997–2016 (men) and 1998–2010 (women). In contrast, both DALYs and ASDR in the United States showed a slow increase over time, except for women whose ASDR declined after 2016. ASDR in Indian men increased over time between 1990 and 2010, then declined abruptly, and showed an upward trend again from 2015 onwards. In contrast, ASDR in Indian women continued to decline after 1998 (Figures 3, **5B**, and Supplementary Table S7). The HCV infection-related deaths and ASMR in the corresponding regions show a similar trend (Supplementary Figure S1, **Figure 5C**, and Supplementary Table S8).

**Figure 4:**
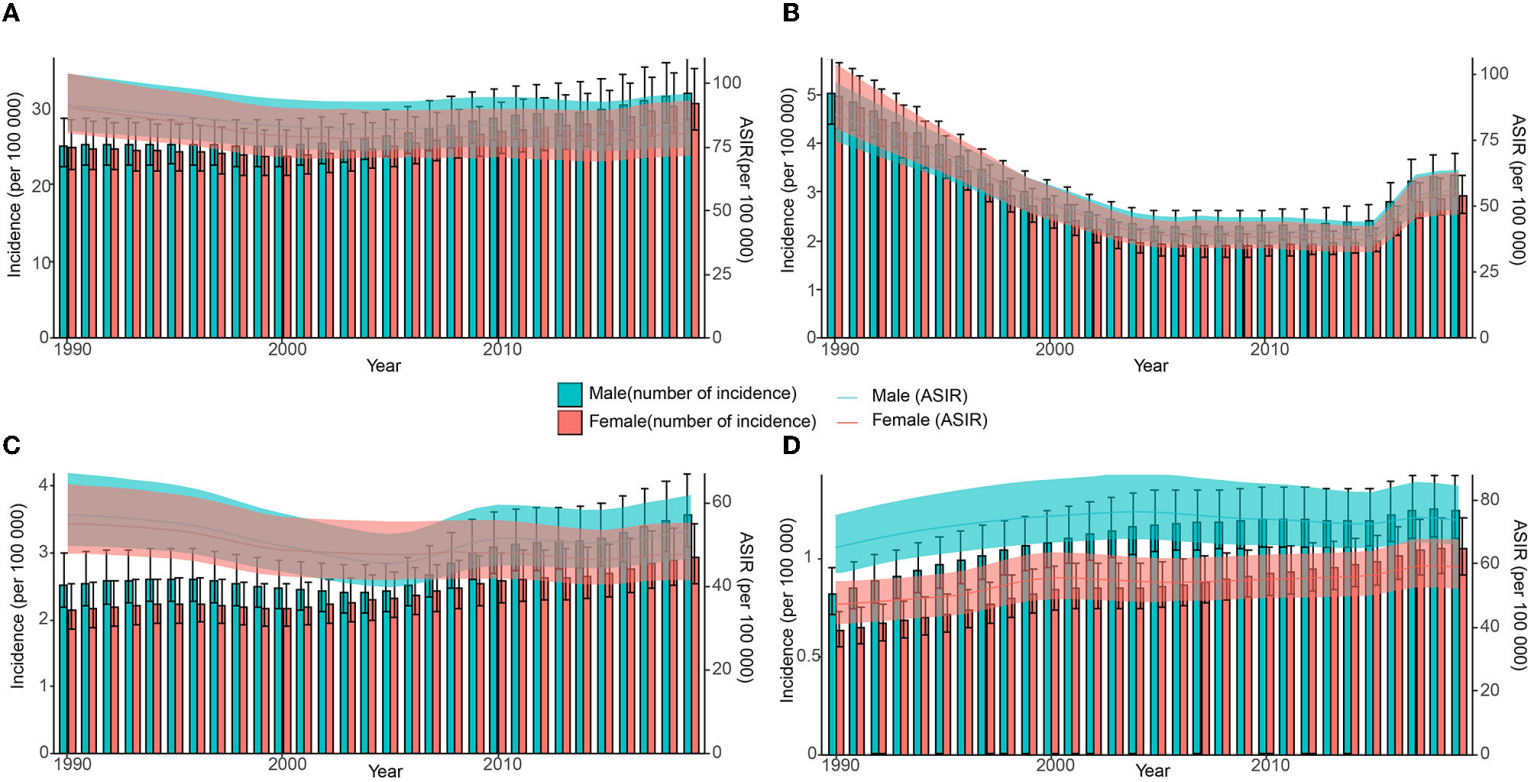
Number and age-standardized rate of incidence with 95% uncertainty intervals (UIs) in different regions, 1990–2019. **(A)** Global; **(B)** China; **(C)** India; **(D)** the United States.

**Figure 5:**
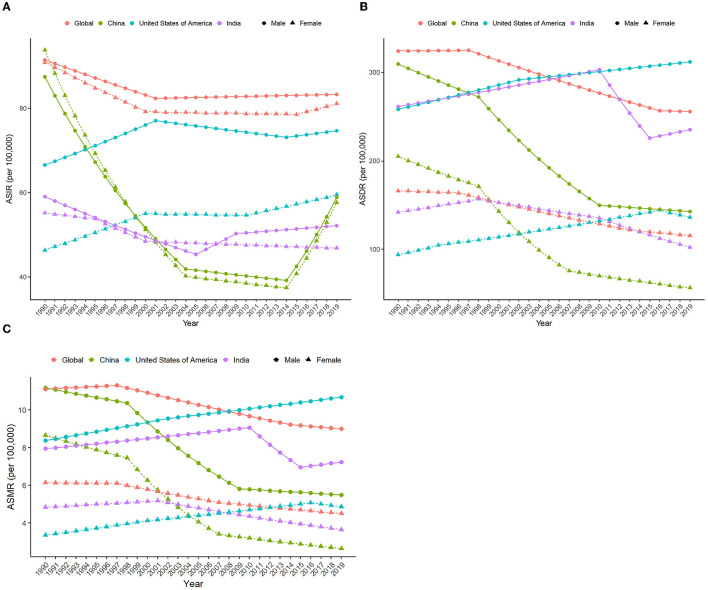
APC in ASIR, ASDR, and ASMR (per 100,000) due to HCV from 1990 to 2019 by sex in different regions. **(A)** ASIR; **(B)** ASDR; **(C)** ASMR. APC, annual percent change; ASIR, age-standardized incidence rate; ASDR, age-standardized DALY rate.

Globally, new HCV infections declined gradually between 1990 and 2000 and then began to slowly increase after 2000. ASIR showed a downward trend until 2001 and has gradually increased since then. The incidence of HCV infection and ASIR in China decreased rapidly in the early stage, then remained relatively flat, and increased rapidly after 2014. The incidence of HCV infection in India is generally similar to the global trend, but the trends in men and women are significantly different. The ASIR of Indian women showed a downward trend from 1990 to 2019, but the ASIR of Indian men declined early, then increased rapidly, and the growth slowed down after 2009. Both HCV infection and ASIR in the United States increased between 1990 and 2019, with women increasing faster than men. Notably, ASIR was similar for men and women globally, in China, and India, whereas ASIR in US men with HCV infection was nearly 1.5 times higher than in women (Figures 4, 5A and Supplementary Table S6).

### 3.3. Age-related difference in the disease burden

**Figure 6:**
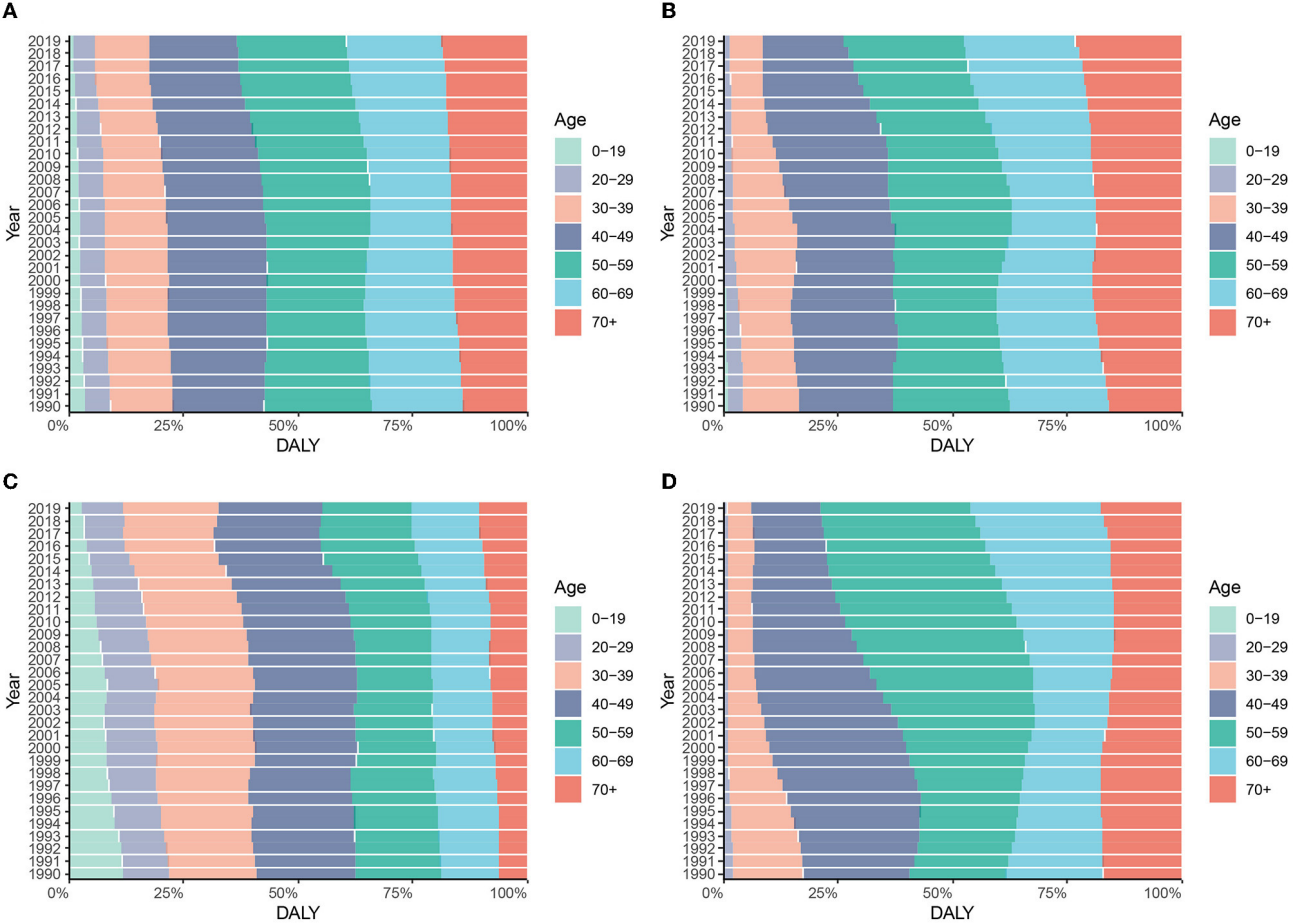
Constitution of DALYs attributable to HCV by age groups in different regions from 1990 to 2019. **(A)** Global; **(B)** China; **(C)** India; **(D)** the United States.

In 2019, the global HCV-related DALYs in people under 60 years of age increased with age, with the highest proportion in the 50–59 age group, and more than half of the 40–69 age group. People over the age of 50 in China account for more than 75% of all DALYs in 2019. The age composition of HCV-related DALYs in the United States is skewed, with the 50–69 age group accounting for more than half of all DALYs. The proportion of DALYs in the age group 0–29 in the United States is significantly lower, while the proportion of people over 60 years old is significantly higher than that in the world, China, and India. In addition, the age composition of HCV infection-related DALYs in the United States showed an aging trend over time, with the most obvious increase in the 50–69 age group. Globally, the age composition of DALYs in China and India changed relatively little between 1990 and 2019 (Figure 6 and Supplementary Figure S2).

In 2019, people over 70 years old accounted for more than 30% of HCV-related deaths in the world, China, and the United States. Among them, the aging population of mortality in the United States is more substantial with the proportion of the 50–69 age group higher than that of the world, China, and India. The age composition ratio of mortality in India is relatively balanced (Supplementary Figures S3, S4).

**Figure 7:**
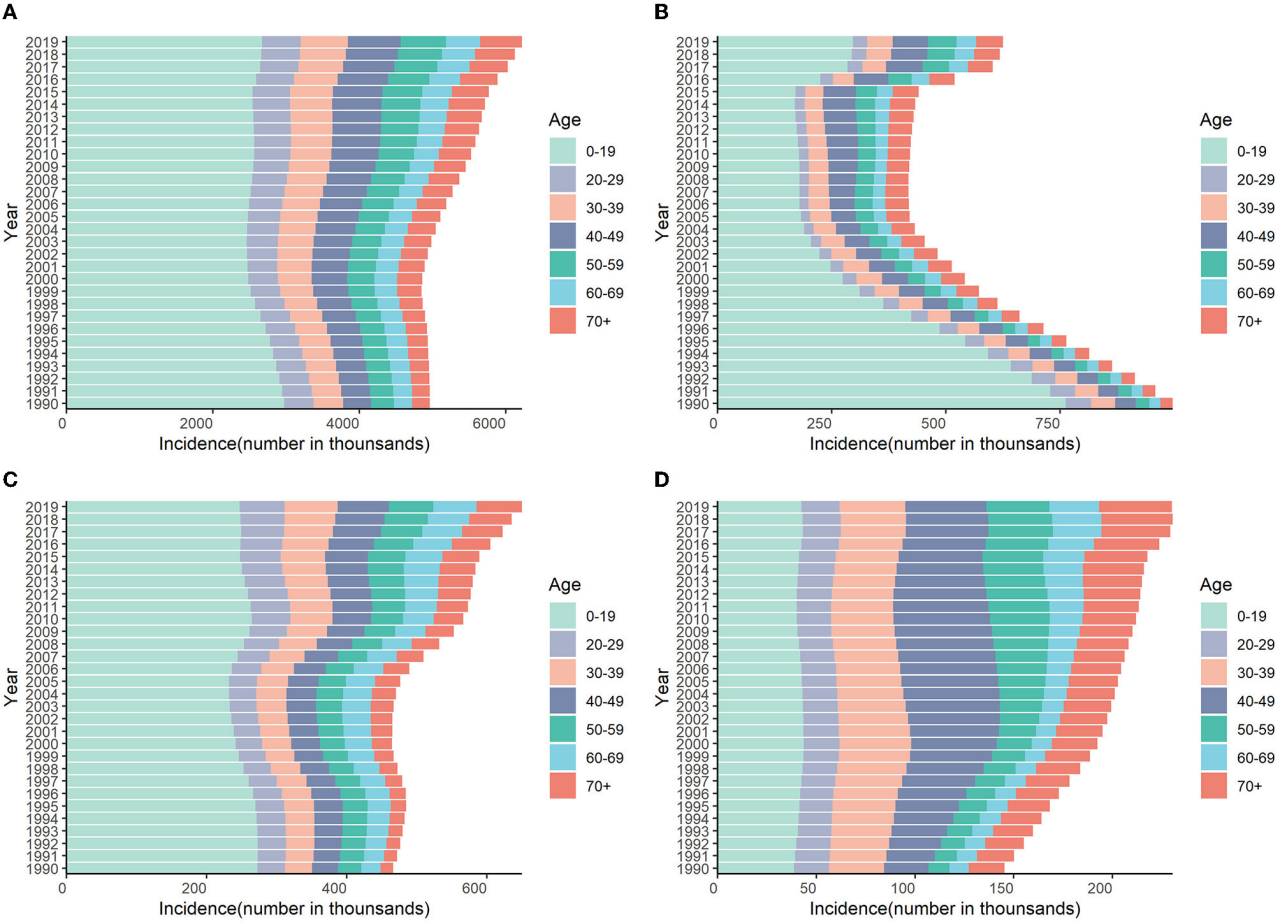
Numbers of incidences attributable to HCV by age groups in different regions from 1990 to 2019. **(A)** Global; **(B)** China; **(C)** India; **(D)** the United States.

The incidence of HCV infection among the 0–19 age group in the world, China, and India accounted for more than half of the total new infections in 2019. The number of cases in China increased rapidly in 2016, mainly in the 0–19 age group. On the contrary, the proportion of people over 40 years old in the United States is significantly higher than that in the world, China, and India (Figure 7 and Supplementary Figure S5).

### 3.4. The burden of HCV by socio-demographic Index (SDI)

**Figure 8:**
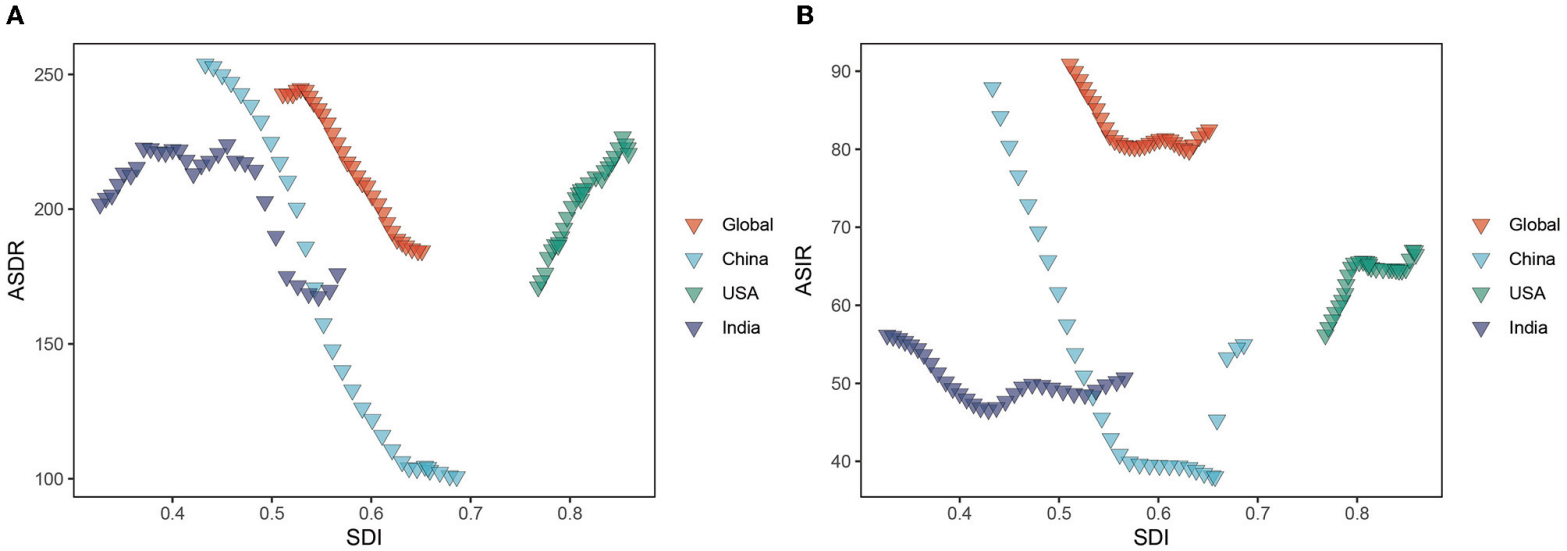
ASDR and ASIR of HCV burden in different regions by SDI from 1990 to 2019. **(A)** ASDR; **(B)** ASIR. ASIR, age-standardized incidence rate; ASDR, age-standardized DALY rate; SDI, Socio-demographic Index.

ASDR and SDI exhibit a non-linear relationship. When SDI is 0.4–0.7, ASDR and SDI are negatively correlated, that is, ASDR decreases with the increase in SDI value, and the disease burden of HCV infection in this interval is higher than other intervals. The largest decrease is found at the SDI between 0.534 and 0.631. However, when the SDI value is less than 0.4 and greater than 0.7, ASDR is positively correlated with SDI, that is, ASDR increases with the increase in SDI. ASDR increases rapidly at 0.768, peaks at 0.853, and then decreases again (Figure 8A). Similar relationships and trends are also found between ASMR and ASIR and SDI. However, ASIR starts to increase with the increase in SDI when SDI is 0.65, stabilizes between SDI 0.80–0.845, and shows an upward trend again after SDI is greater than 0.85 (Figure 8B and Supplementary Figure S6).

### 3.5. Decomposition of changes in disease burden of HCV infection

**Figure 9:**
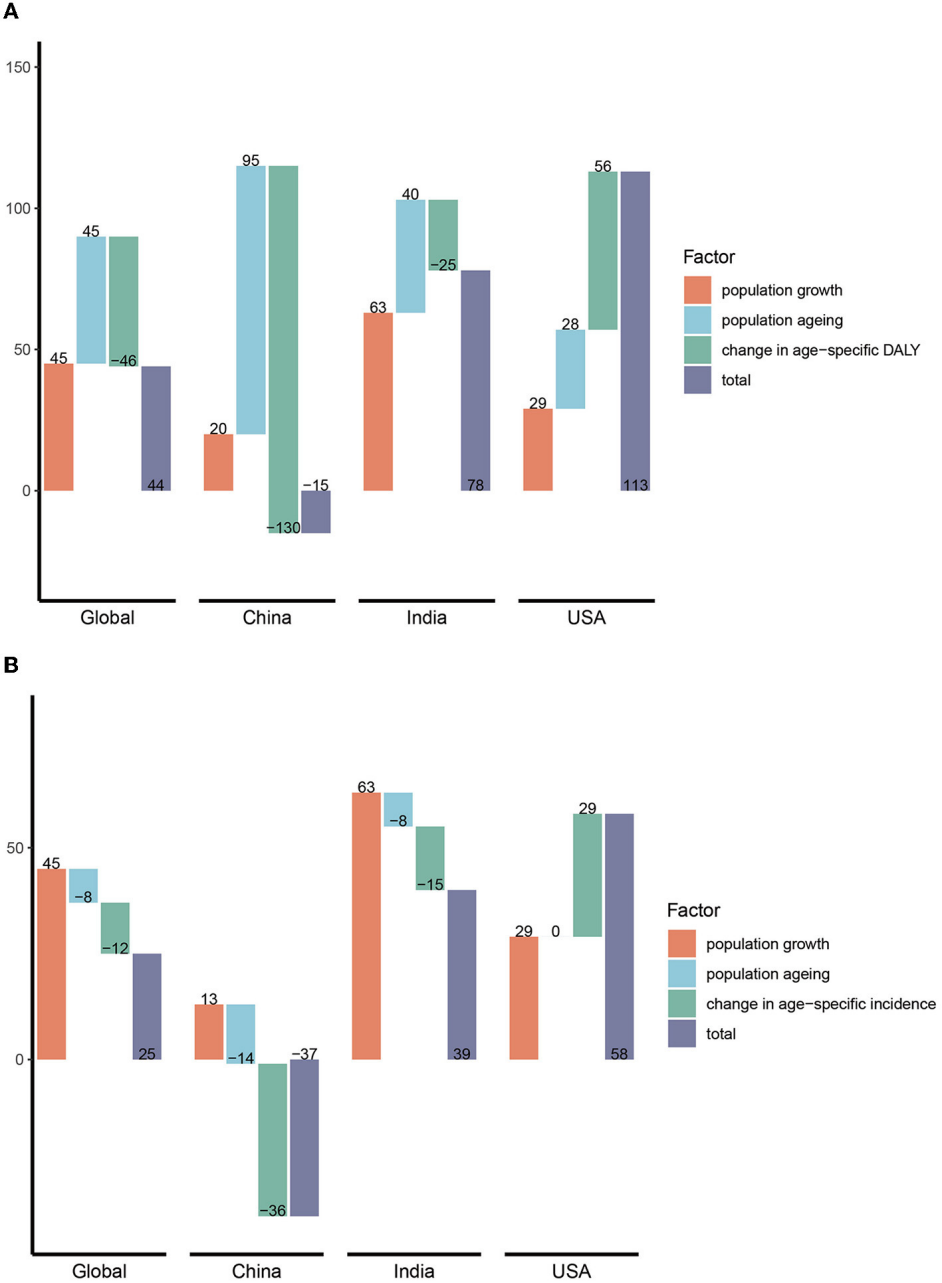
Decomposition of changes (%) in DALY and incidence of HCV in different regions. **(A)** DALY; **(B)** incidence. DALY, disability-adjusted life years.

Between 1990 and 2019, global HCV-related DALY increased by 44% with population growth and population aging each contributing 45% of the increase, while changes in age-specific DALYs offset 46% of the increase. The increase in HCV infection-related DALYs in India was mainly due to population growth (63%), much higher than that of the global, China, and the United States, and the change in age-specific DALYs was the least offset (−25%), resulting in an increase of 78% in DALYs from 1990 to 2019. China's population aging is the main factor for the increase in DALYs, contributing as much as 95%, but because the age-specific DALYs declines more significantly (−130%), the final DALYs in China decreased by 15% compared with 1990. By comparison, population growth, population aging, and age-specific DALY changes have all worsened DALYs in the United States, resulting in as high as 113% growth in DALY, which ranked first among the three countries (Figure 9A). The situation was quite similar in HCV-related death numbers (Supplementary Figure S7).

In terms of incidence, the global population increased by 45%, offset by aging and age-specific changes of 8 and 12%, respectively. As with DALYs, population growth was also a major factor (63%) in the increase in the incidence in India. However, population growth and aging had little impact on the incidence in China, while the decline in age-specific incidence was much greater, offsetting the 36% increase. Finally, the number of new infections in China decreased by 37% compared with 1990. The incidence of HCV infection in the United States has increased significantly due to population and age-specific incidence increases (Figure 9B).

## 4. Discussion

HCV infection is one of the major causes of liver cirrhosis and liver cancer, and its disease burden still tends to increase (9, 16, 32). Although encouraging progress has been made so far, there is still a gap between the 2030 goals. As reported by WHO in 2021, we are not yet on track to erase this infection even without the disturbance of the COVID-19 pandemic (9). The burden of the disease varies across regions and countries, and policy formulation needs to be tailored to local conditions, but in recent years, there are few in-depth and comprehensive studies on the disease burden of HCV infection at the specific country level. Therefore, this study aims to explore the changes in the disease burden of HCV infection in China, India, and the United States from 1990 to 2019, comparing with the global.

According to GBD 2019, the global ASMR and ASDR decreased (mostly in YLL rate) by over 20%, which is consistent with the decreasing trend of ASMR in HCV-related liver cancer and cirrhosis (16, 32). The emergence of DAAs has revolutionized the management of HCV infection, with a cure rate as high as 90% and well-tolerated in various populations (4, 5, 33, 34). Pan-genotypic DAAs have high SVR rates in all six genotypes, which greatly simplify the treatment of HCV infection and improve patient compliance (35, 36). In addition, the price of DAAs has dropped significantly, improving access to patients in less economically developed regions. Data show that from 2015 to the end of 2019, 9.5 million patients with HCV received antiviral treatment, of which 8.7 million (8.5–10.04 million) were cured (9, 17). In addition, the reduction in HCV diagnostic costs and the optimization of diagnostic methods also provide a great convenience for large-scale screening of HCV infection (9, 37).

China's ASRs showed an overall downward trend, and the decline was greater than that of India and the global, while the ASRs of the United States showed an upward trend. According to the study of Polaris HCV Collaborative Group, more than 1 million patients in the United States, 790,000 patients in China, and 630,000 patients in India initiated antiviral treatment from 2015 to the end of 2019 (17). Compared with the HCV-infected patient bases in China and India, the number of treated patients is insufficient. The findings were estimated from data reported by public authorities such as drug suppliers and government reports, so the true figures may be underestimated. Although DAAs in China were not approved for marketing until 2017, patients had already self-purchased generic drugs for treatment before then. In 2020, the price of DAAs in the Chinese market was further reduced, and they were included in the national social medical insurance, thus allowing more patients with HCV to receive treatment. Advances in medical care and strict screening system for blood products may be the main reason for the sharp decline of HCV infection in early periods in China (38, 39). It is worth noting that the prevalence of HCV in Chinese women is higher than that in India and the United States, which partially explains the lower disease burden in China because male patients infected with HCV have a higher risk of liver cirrhosis and HCC (20, 33, 40). Meanwhile, according to the deconstructive analysis, population growth and population aging were the main reasons for the increase in HCV-related deaths and DALYs while the decrease was mainly attributed to changes in age-specific deaths and DALYs.

On the contrary, the United States had the highest ASMR and ASDR of the three countries, with greater growth in women than men. Patients with HCV infection in the United States are mainly men, concentrated in middle-aged and elderly people according to our age structure analysis, probably because of the baby boomers. This may explain their higher ASMR and ASDR as male and older age both affect the prognosis of liver cirrhosis and liver cancer (16, 32, 41). In addition, decomposition analysis also found that the increase in DALYs and deaths related to HCV infection in the United States was mainly due to the increase in age-specific DALYs and deaths, which was almost equal to the sum of the contributions of population growth and aging. This may be due to suboptimal treatment rates for HCV infection in the United States. Studies have found that younger patients (18–29 years) have lower treatment rates than older patients (50–59 years) and that patients with commercial insurance have higher rates of treatment than those on Medicaid (42–44). In addition, patients with a history of drug use, patients infected with HIV, and patients with decompensated cirrhosis are also less likely to receive treatment (43, 45). Of note, intravenous drug use is the main factor of HCV infection in the United States with genotype 3 dominated (45), and genotype 3, especially genotype 3b patients with liver cirrhosis, has a significantly lower success rate of DAAs treatment than other genotypes (46, 47). Studies have also shown that IDUs in the United States are generally older, and the proportion of women is higher than that of other low-income countries (48), which may explain the rapid increase in the burden of HCV infection among U.S. women. Furthermore, some patients are still at risk of developing HCC even after HCV was cured (49, 50). Younosssi et al. (22) found that cirrhosis and liver cancer in the United States are still on the rise, of which HCV infection is the main cause, and the disease burden caused by alcoholic liver disease (ALD) and non-alcoholic fatty liver diseases (NAFLD) is also increasing. Patients with HCV in the United States may be older and have a long history of infection, and most have advanced fibrosis or cirrhosis. They may also have ALD or NAFLD at the same time, which aggravates the progression of liver diseases. The COVID-19 epidemic has further hindered the management of HCV infection, so the data after 2020 may be even more unsatisfactory (51).

The ASMR and ASDR of Indian male patients showed a trend of increasing first, declining rapidly, and then increasing, while the ASMR and ASDR of Indian female patients had a downward trend in the past 20 years. The age composition of patients infected with HCV in India is younger than that in China and the United States. This is consistent with a previous study in which HCV infection in India was concentrated among middle-aged men (41–60 years) in rural areas. The main three risk factors for HCV infection in India are dental treatment, injection treatment with non-disposable needles and syringes, and body piercing (52). Another study also found that injection treatment and blood transfusion are the main risk factors for the transmission of HCV infection in India, and patients were mainly genotype 3 which was associated with worse prognosis (52–54). Therefore, improving medical conditions is an important measure that India should take, especially the replacement of reused needles and syringes with disposable needles and syringes. Drug users are less common in India than in developed countries such as the United States, which explained the middle-age-dominated age structure in India (52, 53).

A systematic review reported nearly half of new HCV infections worldwide occurred in IDUs (48), and intravenous drug use was an important risk factor for new and repeated infection. Since 2015, ASIR of HCV infection has increased more rapidly in women than in men globally, which is consistent with previous studies (15). Compared with China and India, the United States had the highest ASIR in 2019, and the ASIR of male patients in the United States was significantly higher than that of women, which may be because the main risk factor for HCV infection in the United States is intravenous drug use, and drug users are mainly men (45, 48). ASIR among Indian women has gradually declined since 1990 probably because 99% of HCV infections of IDUs were young men (55).

It should be noted that the increase in the number of cases may be also due to the improvement of diagnostic technology and the expansion of screening, leading to the discovery of originally infected persons, or the improvement of the infectious disease reporting system that promotes the reporting of more cases (40). Moreover, an increase in drug use and sexual transmission may be also responsible for the increase in cases according to our previous study (39).

The highest and lowest ASDR and ASMR in HCV disease burden occurred at SDI values of 0.433 and 0.686, respectively. They were negatively correlated at SDI values of 0.4-0.7 and positively correlated at values less than 0.4 and greater than 0.7. ASDR and SDI in acute HCV infection and HCC also had a similar U-shaped relationship (15, 16). This contradictory situation was probably because the United States had many IDUs while unsafe medical treatment remained a major problem in India, that is, the disease burden will increase in both high and low-economic development countries, although the high-risk groups and transmission routes may be different.

The strengths of this study are as follows. First, this study applied the results of GBD data, which are currently the only comprehensive estimate of the etiology-specific disease burden of 359 diseases and injuries in 204 countries and regions around the world, covering the period from 1990 to 2019, which can better fully explore the disease burden from all levels (24). Second, we applied joinpoint analysis to model the temporal trends and turning points, which can clarify the temporal trends from a statistical point of view. Finally, this study made an in-depth comparison of the disease burden among countries with different economic development levels, in terms of gender, age, and time-varying trends, and further analyzed the contribution of population growth, population aging, and age-specific changes to the change in cases.

However, our analysis has limitations, too. First, the GBD data are estimates, and the accuracy of the estimates relied highly on the quality of the data. The data quality of the three countries involved in this study and the whole world was relatively high during the study period, and the GBD was estimated by a very reliable model (22) (Supplementary Table S5). Second, the disease burden of HCV infection may still be underestimated, because HCV infection is mostly asymptomatic, and the awareness rate of the disease is extremely low (9). Finally, the data lack information on risk factors, so further research on the risk factors is needed in future.

## 5. Conclusion

In summary, although gratifying progress has been made in the management of HCV, it remains a worldwide challenge faced by both developed countries and developing countries (9). Together with the interruption of the COVID-19 pandemic, accelerated collective efforts are required to approach the 2030 goal. Our study provides information for policymakers to implement tailored strategies. China should expand the number of people diagnosed and treated, especially in economically underdeveloped areas while the United States should focus on key populations and try to improve patient outcomes. India, however, should provide disposable needles, safe blood, and dental treatment, especially in rural regions.

## Data availability statement

The datasets presented in this study can be found in online repositories. The names of the repository/repositories and accession number(s) can be found below: https://ghdx.healthdata.org/gbd-results-tool.

## Author contributions

JY, H-XL, and H-YR: study design and oversight of study. JY and H-XL: data collection, data analysis, and interpretation. JY: drafting of the manuscript. H-XL and J-LQ: provide methodological guidance. X-XW, X-HL, RJ, and B-YL: participate in interpretation of results. All authors review and approval of final manuscript.
