# Supplementary material for: The burden of hepatitis C virus in the world, China, India, and the United States from 1990 to 2019
Source: Front Public Health. 2023 Mar 2;11:1041201. doi: 10.3389/fpubh.2023.1041201 (PMC10018168; doi:10.3389/fpubh.2023.1041201)
Supplement: Supplementary file 1 [file Data_Sheet_1.pdf]

# Supplementary Material

## SUPPLEMENTARY TABLES AND FIGURES

## TABLES

Table S1. Codes for definitions of Total burden related to hepatitis C in GBD 2019

| Total burden related to hepatitis C | ICD10 | ICD9 |
|---|---|---|
| Acute hepatitis C | B15-B16.2, 17.0, B17.2, B19.1, P35.3 | 070.0-070.2 |
| Cirrhosis and other chronic liver diseases due to hepatitis C | B18-B18.9, I85-I85.9, I98.2, K70-K70.3, K71.7, K73-K75, K75.2, K75.4-K76.2, K76.4-K76.9, K77.8 | 456.0-456.2 571-571.9, 572.2-573.0, 573.4-573.9 |
| Liver cancer due to hepatitis C | C22-C22.8, D13.4 | 155-155.1, 155.3-155.9, 211.5 |

Table S2. Mortality, age-standardized rates, and their temporal trends of HCV infection in different regions, 1990 vs.2019

| Country | Sex | Mortality | | | | | |
|---|---|---|---|---|---|---|---|
| | | Number in thousands 95%UI | | Percentage change in number (95% UI) | Age-standardized rate per 100,000 95%UI | | Percentage change in rate (95% UI) |
| | | 1990 | 2019 | 1990-2019 | 1990 | 2019 | 1990-2019 |
| Global | Overall | 340.9<br>(300.7∼381.6) | 542.3<br>(476.7∼608.9) | 59.1%<br>(46.9%∼73.3%) | 8.5<br>(7.5∼9.4) | 6.7<br>(5.9∼7.5) | -21.1%<br>(-27%∼-14.4%) |
| | Male | 210.8<br>(183.3∼238.6) | 344.9<br>(301.1∼392.2) | 63.6%<br>(49.5%∼81%) | 11.1<br>(9.7∼12.5) | 9<br>(7.9∼10.2) | -18.6%<br>(-25.3%∼-10.3%) |
| | Female | 130.1<br>(114.1∼148.2) | 197.4<br>(170.3∼223.4) | 51.8%<br>(35.6%∼70.9%) | 6.1<br>(5.4∼6.9) | 4.5<br>(3.9∼5.1) | -25.6%<br>(-32.9%∼-16.8%) |
| China | Overall | 80<br>(68.2∼92.8) | 78<br>(64.4∼92.1) | -2.4%<br>(-20.7%∼20.5%) | 9.7<br>(8.4∼11.2) | 4<br>(3.4∼4.7) | -58.5%<br>(-65.7%∼-49.4%) |
| | Male | 45.5<br>(36.9∼54.8) | 49.6<br>(38.6∼60.7) | 9%<br>(-18.1%∼47%) | 11<br>(9∼13) | 5.5<br>(4.3∼6.6) | -50%<br>(-61.7%∼-34.1%) |
| | Female | 34.5<br>(28.3∼42.4) | 28.4<br>(22.5∼35.2) | -17.6%<br>(-38.5%∼10.9%) | 8.4<br>(6.9∼10.2) | 2.8<br>(2.2∼3.5) | -66.8%<br>(-74.8%∼-55.7%) |
| India | Overall | 32.9<br>(27.3∼41.4) | 66.7<br>(54.7∼80.7) | 102.7%<br>(65.5%∼148.4%) | 6.3<br>(5.3∼7.9) | 5.6<br>(4.6∼6.7) | -11.3%<br>(-6.5%∼7.4%) |
| | Male | 21.7<br>(17.9∼27.1) | 44.1<br>(34.7∼54.9) | 103%<br>(57.9%∼160.6%) | 7.7<br>(6.3∼9.6) | 7.3<br>(5.8∼9.1) | -5.2%<br>(-26.1%∼21.3%) |
| | Female | 11.2<br>(8.2∼15.9) | 22.6<br>(16.9∼29.7) | 102%<br>(43.9%∼177.2%) | 4.7<br>(3.6∼6.4) | 3.9<br>(2.9∼5.1) | -18.4%<br>(-39.3%∼9.5%) |
| America | Overall | 17<br>(15.4∼18.7) | 39.2<br>(35.5∼43.1) | 131%<br>(121.2%∼140.6%) | 5.6<br>(5.1∼6.2) | 7.6<br>(7∼8.4) | 35.2%<br>(29.9%∼40.2%) |
| | Male | 11.1<br>(10.1∼12.2) | 25.4<br>(22.9∼28.1) | 128.5%<br>(116.5%∼140.5%) | 8.4<br>(7.6∼9.2) | 10.6<br>(9.6∼11.7) | 26.8%<br>(20.7%∼32.8%) |
| | Female | 5.8<br>(5.2∼6.6) | 13.7<br>(12.1∼15.3) | 135.9%<br>(123.1%∼153%) | 3.3<br>(3∼3.7) | 4.9<br>(4.4∼5.4) | 47.2%<br>(40.2%∼56.7%) |

Table S3. Socio-demographic index in the world and three countries from 1990 to 2019

| Location | 1990 | 1991 | 1992 | 1993 | 1994 | 1995 | 1996 | 1997 | 1998 | 1999 | 2000 | 2001 | 2002 | 2003 | 2004 | 2005 | 2006 | 2007 | 2008 | 2009 | 2010 | 2011 | 2012 | 2013 | 2014 | 2015 | 2016 | 2017 | 2018 | 2019 |
|---|---|---|---|---|---|---|---|---|---|---|---|---|---|---|---|---|---|---|---|---|---|---|---|---|---|---|---|---|---|---|
| Global | 0.511 | 0.516 | 0.521 | 0.525 | 0.529 | 0.534 | 0.538 | 0.542 | 0.547 | 0.551 | 0.556 | 0.561 | 0.566 | 0.571 | 0.576 | 0.581 | 0.586 | 0.591 | 0.596 | 0.601 | 0.607 | 0.612 | 0.616 | 0.621 | 0.626 | 0.631 | 0.635 | 0.641 | 0.647 | 0.651 |
| China | 0.433 | 0.441 | 0.45 | 0.459 | 0.469 | 0.479 | 0.489 | 0.499 | 0.508 | 0.516 | 0.525 | 0.534 | 0.543 | 0.552 | 0.561 | 0.571 | 0.581 | 0.591 | 0.601 | 0.611 | 0.621 | 0.631 | 0.638 | 0.646 | 0.654 | 0.657 | 0.659 | 0.669 | 0.679 | 0.686 |
| USA | 0.768 | 0.771 | 0.775 | 0.778 | 0.782 | 0.785 | 0.788 | 0.789 | 0.791 | 0.794 | 0.797 | 0.802 | 0.806 | 0.809 | 0.811 | 0.812 | 0.811 | 0.814 | 0.819 | 0.826 | 0.832 | 0.835 | 0.839 | 0.842 | 0.845 | 0.849 | 0.853 | 0.856 | 0.858 | 0.859 |
| India | 0.327 | 0.333 | 0.339 | 0.345 | 0.351 | 0.358 | 0.364 | 0.371 | 0.378 | 0.386 | 0.393 | 0.4 | 0.407 | 0.414 | 0.421 | 0.429 | 0.437 | 0.446 | 0.455 | 0.463 | 0.473 | 0.483 | 0.493 | 0.504 | 0.515 | 0.526 | 0.537 | 0.547 | 0.558 | 0.566 |

Table S4. SDI Reference Quintiles(Updated on March 21, 2021, with an SDI reference quintiles file. These quintiles provide a reference for the categorization between locations with Low, Low-Middle, Middle, Highmiddle, and High SDI.)

| sdi_quintile | lower_bound | upper_bound |
|---|---|---|
| Low SDI | 0 | 0.454743 |
| Low-middle SDI | 0.454743 | 0.607679 |
| Middle SDI | 0.607679 | 0.689504 |
| High-middle SDI | 0.689504 | 0.805129 |
| High SDI | 0.805129 | 1 |

Table S5. Country list of GBD Geographic With Data Quality Rating for Causes of Death.Data Star-rating system based on the proportion of death registered to a well-defined cause (percent well-certified): 5 stars: 85%–100% well-certified; 4 stars: 65%–84%, 3 stars: 35%–64%, 2 stars: 10%–34%, 1 star: >0%–9%, 0 stars: No VR or VA data available from 2010–2019

| Country | Data Quality Rating | 1980-1984 | 1985-1989 | 1990-1994 | 1995-1999 | 2000-2004 | 2005-2009 | 2010-2019 | 1980-2019 |
|---|---|---|---|---|---|---|---|---|---|
| China | 3 | 0.0 | 1.0 | 70.0 | 73.7 | 66.8 | 72.1 | 71.7 | 50.8 |
| India | 2 | 2.6 | 4.6 | 2.7 | 4.8 | 6.3 | 49.7 | 46.0 | 16.7 |
| USA | 5 | 90.4 | 89.6 | 90.1 | 89.5 | 88.6 | 87.8 | 87.2 | 89.0 |

Table S6. APC and 95% CI in ASIR (per 100 000) due to HCV from 1990 to 2019 by sex. APC, annual percent change; ASIR, age-standardized incidence rate; CI, confidence interval.

| | Global | | China | | India | | USA | |
|---|---|---|---|---|---|---|---|---|
| Sex | Year | APC(95% CI) | Year | APC(95% CI) | Year | APC 95% CI) | Year | APC(95% CI) |
| Male | 1990-2001 | -0.95*(-1.08~-0.82) | 1990-2004 | -5.12*(-5.47~-4.78) | 1990-2005 | -1.74*(-1.93~-1.55) | 1990-2001 | 1.34*(1.19~1.5) |
| | 2001-2019 | 0.06*(0~0.12) | 2004-2014 | -0.66(-1.36~0.04) | 2005-2009 | 2.61*(0.39~4.87) | 2001-2014 | -0.4*(-0.53~-0.27) |
| | 1990-2019 | -0.32*(-0.38~-0.26) | 2014-2019 | 8.48*(6.55~10.44) | 2009-2019 | 0.37*(0.03~0.72) | 2014-2019 | 0.42(-0.05~0.89) |
| | | | 1990-2019 | -1.35*(-1.76~-0.95) | 1990-2019 | -0.43*(-0.74~-0.11) | 1990-2019 | 0.4*(0.29~0.51) |
| Female | 1990-2000 | -1.37*(-1.44~-1.3) | 1990-2004 | -5.87*(-6.24~-5.51) | 1990-1995 | -0.53(-1.08~0.03) | 1990-2000 | 1.73*(1.47~1.99) |
| | 2000-2015 | -0.05*(-0.09~-0.01) | 2004-2014 | -0.7(-1.47~0.07) | 1995-2000 | -2.06*(-2.81~-1.3) | 2000-2010 | -0.07(-0.35~0.2) |
| | 2015-2019 | 0.78*(0.49~1.07) | 2014-2019 | 9.04*(6.86~11.27) | 2000-2019 | -0.18*(-0.25~-0.11) | 2010-2019 | 0.96*(0.68~1.23) |
| | 1990-2019 | -0.4*(-0.44~-0.35) | 1990-2019 | -1.66*(-2.1~-1.22) | 1990-2019 | -0.56*(-0.72~-0.41) | 1990-2019 | 0.86*(0.72~1.01) |

*p <0.05

Table S7. APC and 95% CI in ASDR (per 100 000) due to HCV from 1990 to 2019 by sex. APC, annual percent change; ASDR, age-standardized DALY rate; CI, confidence interval.

| | Global | | China | | India | | USA | |
|---|---|---|---|---|---|---|---|---|
| Sex | Year | APC (95% CI) | Year | APC (95% CI) | Year | APC (95% CI) | Year | APC (95% CI) |
| Male | 1990-1997 | 0.04(-0.19~0.28) | 1990-1998 | -1.59*(-1.98~-1.2) | 1990-2010 | 0.74*(0.59~0.9) | 1990-2002 | 1.02*(0.84~1.2) |
| | 1997-2016 | -1.24*(-1.29~-1.18) | 1998-2010 | -4.85*(-5.05~-4.65) | 2010-2015 | -5.72*(-7.09~-4.33) | 2002-2019 | 0.4*(0.29~0.5) |
| | 2016-2019 | -0.12(-1.08~0.86) | 2010-2019 | -0.54*(-0.91~-0.16) | 2015-2019 | 1.03(-0.88~2.98) | 1990-2019 | 0.65*(0.56~0.74) |
| | 1990-2019 | -0.81*(-0.93~-0.7) | 1990-2019 | -2.63*(-2.8~-2.46) | 1990-2019 | -0.36*(-0.72~0) | | |
| Female | 1990-1996 | -0.22(-0.53~0.08) | 1990-1998 | -2.23*(-3.25~-1.2) | 1990-1998 | 1.23*(0.26~2.2) | 1990-1994 | 2.79*(1.82~3.77) |
| | 1996-2013 | -1.73*(-1.78~-1.67) | 1998-2007 | -8.68*(-9.41~-7.94) | 1998-2010 | -1.19*(-1.61~-0.77) | 1994-2016 | 1.47*(1.4~1.53) |
| | 2013-2019 | -0.87*(-1.15~-0.6) | 2007-2019 | -2.4*(-2.93~-1.86) | 2010-2019 | -3.07*(-3.63~-2.51) | 2016-2019 | -1.86*(-3.34~-0.36) |
| | 1990-2019 | -1.24*(-1.33~-1.15) | 1990-2019 | -4.34*(-4.75~-3.94) | 1990-2019 | -1.12*(-1.46~-0.78) | 1990-2019 | 1.3*(1.1~1.5) |

*p <0.05

Table S8. APC and 95% CI in ASMR (per 100 000) due to HCV from 1990 to 2019 by sex. APC, annual percent change; ASMR, age-standardized mortality rate; CI, confidence interval.

| | Global | | China | | India USA | | | |
|---|---|---|---|---|---|---|---|---|
| Sex | Year | APC (95% CI) | Year | APC (95% CI) | Year | APC (95% CI) | Year | APC (95% CI) |
| Male | 1990-1997 | 0.26*(0.14~0.38) | 1990-1998 | -0.92*(-1.48~-0.36) | 1990-2010 | 0.66*(0.51~0.81) | 1990-2002 | 1.09*(0.94~1.25) |
| | 1997-2014 | -1.2*(-1.23~-1.17) | 1998-2009 | -5.12*(-5.44~-4.8) | 2010-2015 | -5.15*(-6.53~-3.74) | 2002-2019 | 0.66*(0.57~0.76) |
| | 2014-2019 | -0.48*(-0.68~-0.28) | 2009-2019 | -0.57*(-1~-0.14) | 2015-2019 | 0.99(-0.88~2.9) | 1990-2019 | 0.84*(0.76~0.92) |
| | 1990-2019 | -0.72*(-0.77~-0.68) | 1990-2019 | -2.42*(-2.65~-2.18) | 1990-2019 | -0.32(-0.67~0.04) | | |
| Female | 1990-1997 | -0.09(-0.31~0.12) | 1990-1998 | -1.84*(-2.81~-0.87) | 1990-2001 | 0.62*(0.1~1.16) | 1990-2000 | 2.05*(1.85~2.25) |
| | 1997-2007 | -1.78*(-1.92~-1.65) | 1998-2007 | -8.33*(-9.04~-7.61) | 2001-2019 | -1.92*(-2.13~-1.71) | 2000-2016 | 1.31*(1.21~1.41) |
| | 2007-2019 | -1.03*(-1.13~-0.94) | 2007-2019 | -2.09*(-2.59~-1.58) | 1990-2019 | -0.96*(-1.19~-0.74) | 2016-2019 | -1.4*(-2.72~-0.07) |
| | 1990-2019 | -1.07*(-1.14~-0.99) | 1990-2019 | -4*(-4.39~-3.62) | | | 1990-2019 | 1.28*(1.12~1.44) |

*p <0.05

# FIGURES

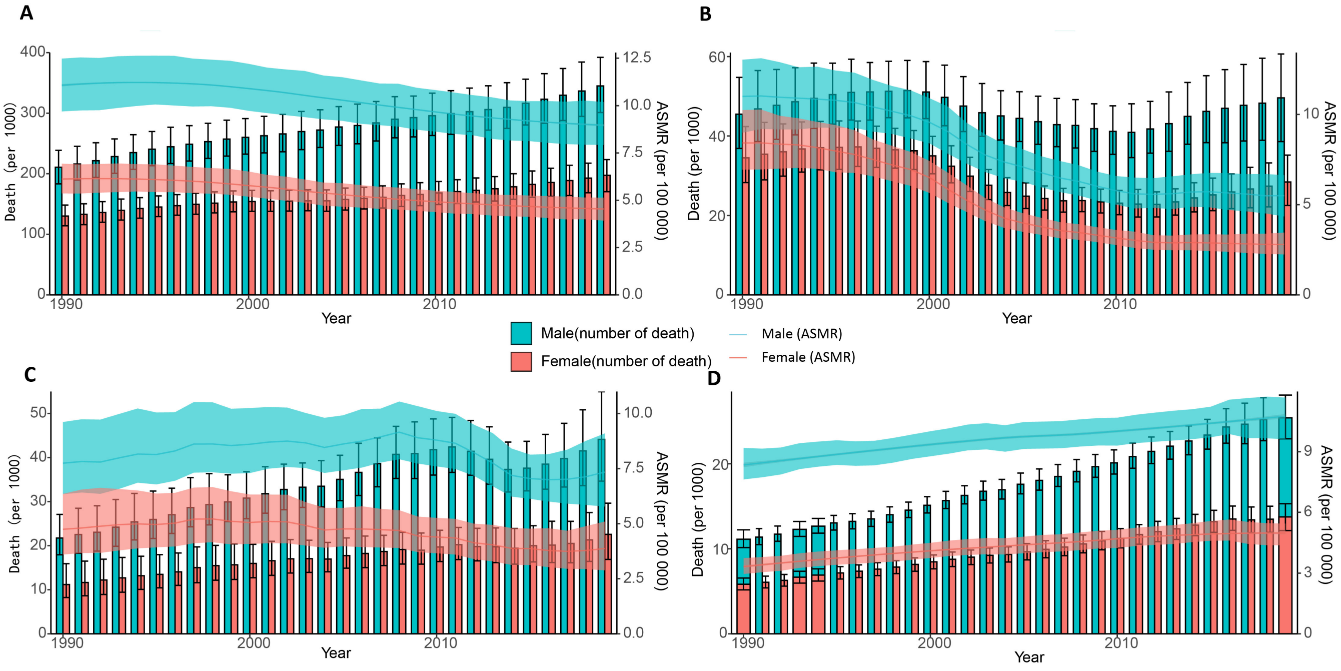


Figure S1. Number and age-standardized rate of mortality in different regions, 1990-2019.(A) Global;(B) China; (C) India; (D) The United States.

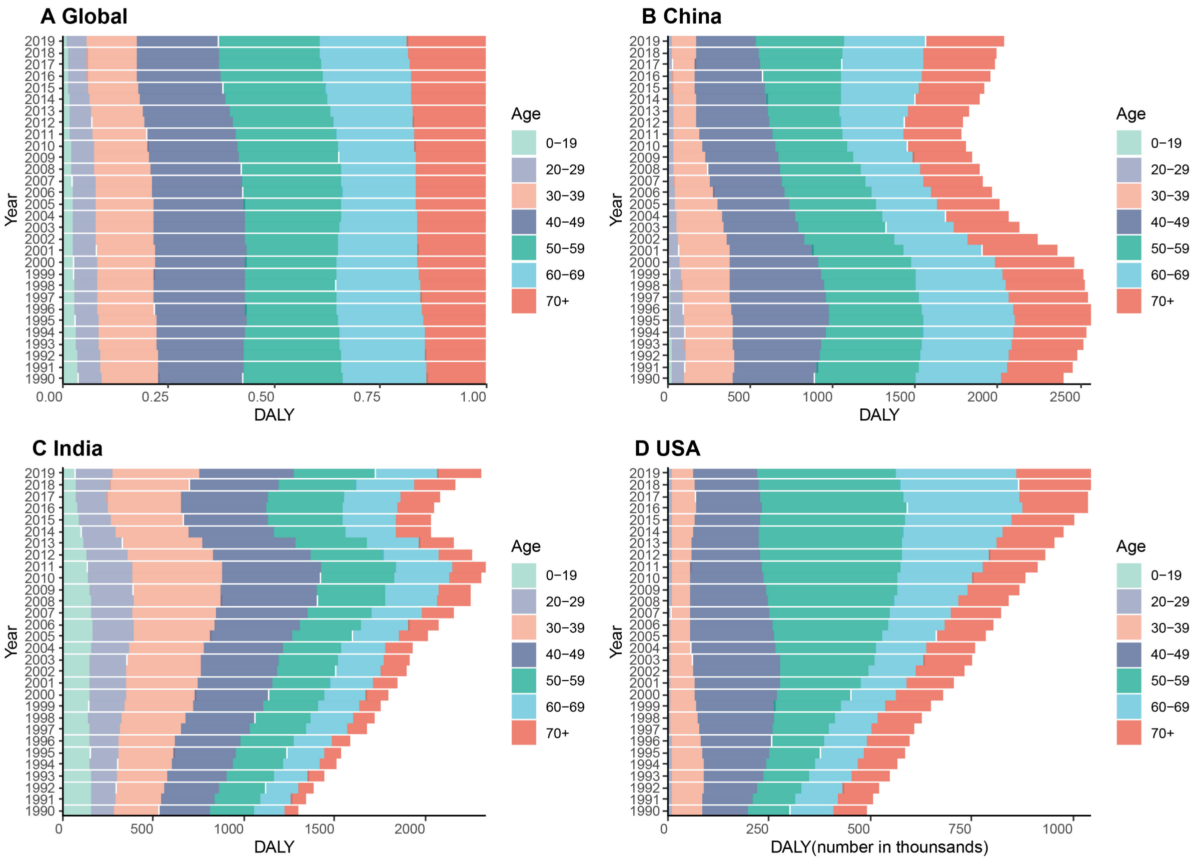


Figure S2. Numbers of DALYs attributable to HCV by age group in different regions from 1990 to 2019.(A) Global; (B) China; (C) India; (D) The United States.

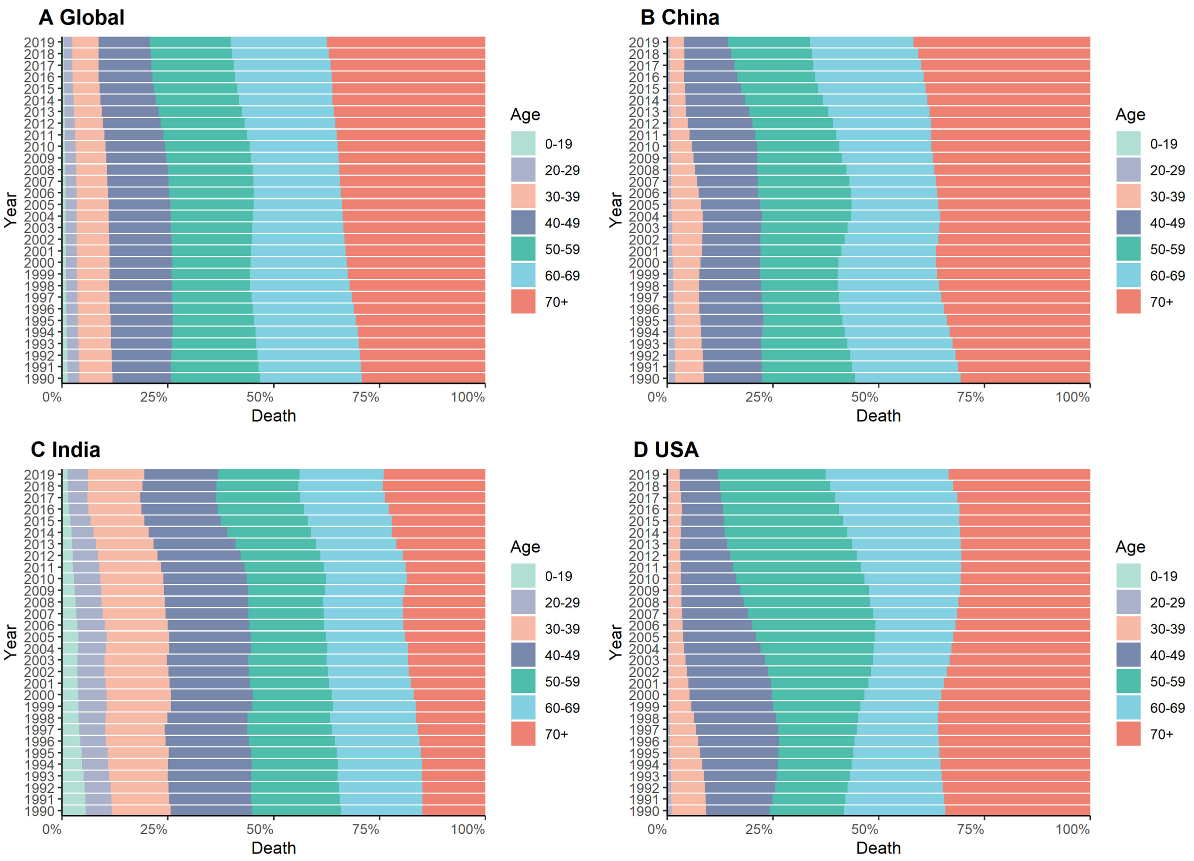


Figure S3. Constitution of death attributable to HCV by age groups in different regions from 1990 to 2019.(A) Global; (B) China; (C) India; (D) The United States.

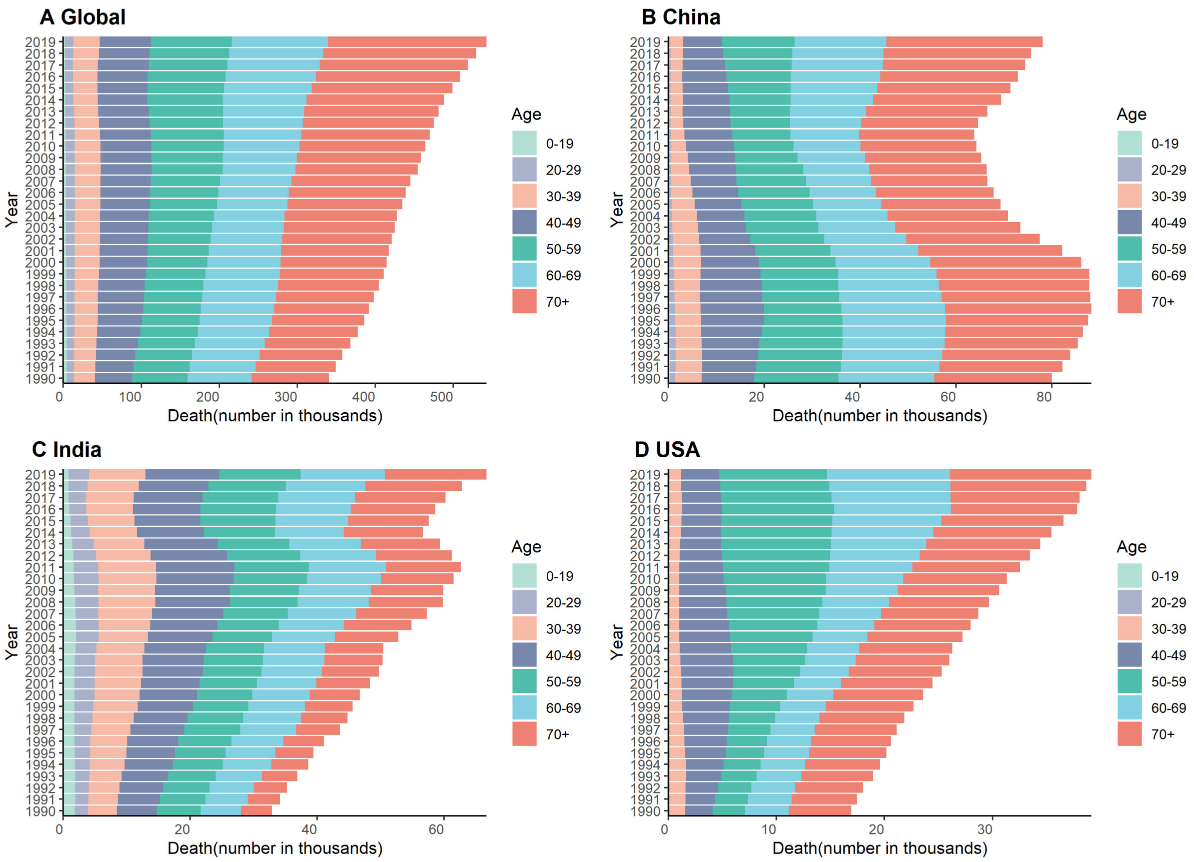


Figure S4. Numbers of death attributable to HCV by age groups in different regions from 1990 to 2019.(A) Global; (B) China; (C) India; (D) The United States.

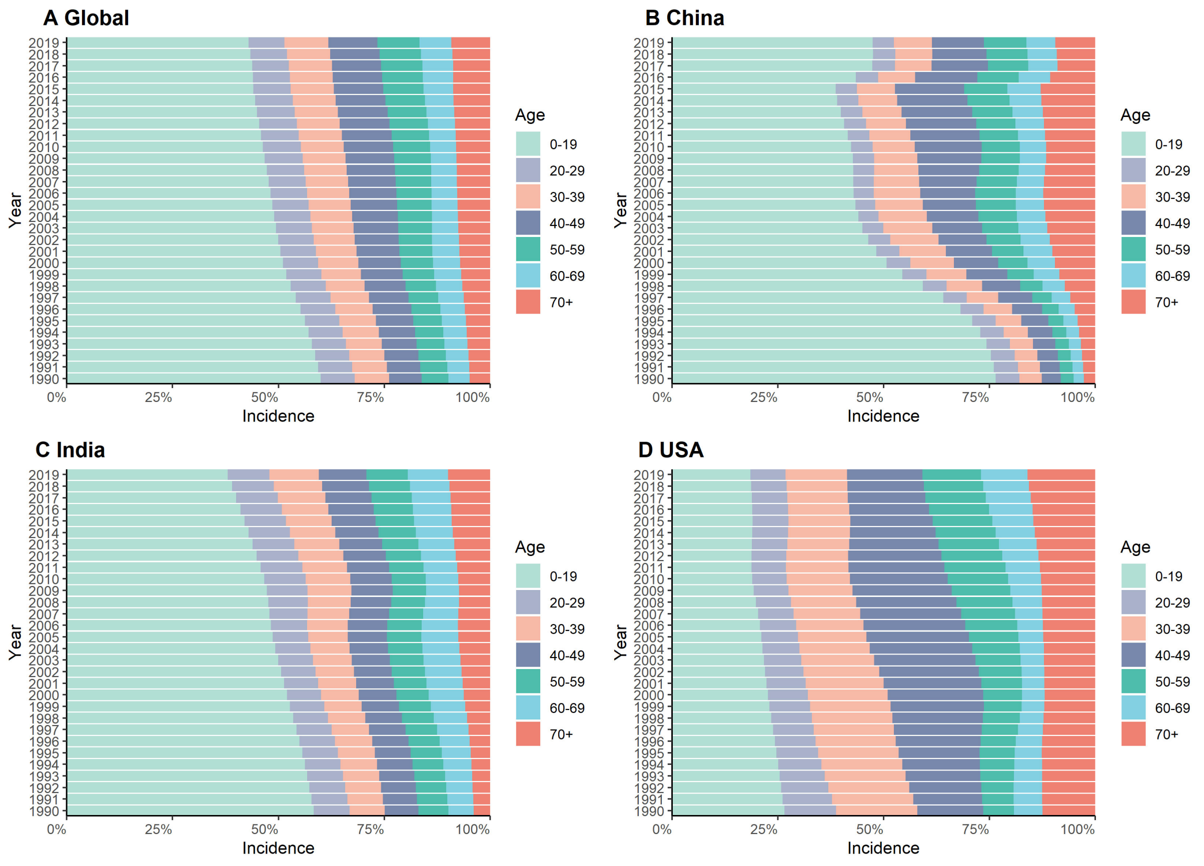


Figure S5. Constitution of incidences attributable to HCV by age groups in different regions from 1990 to 2019.(A) Global; (B) China; (C) India; (D) The United States.

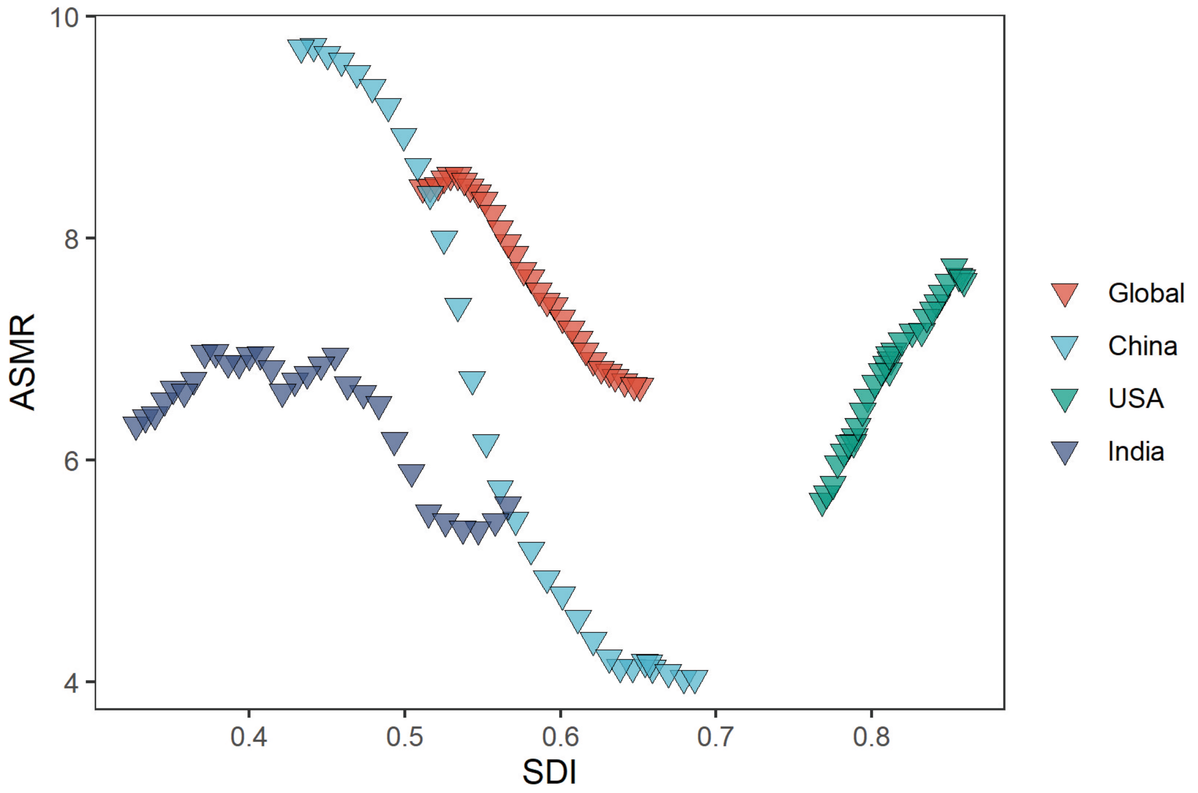


Figure S6. ASMR of HCV burden in different regions by SDI from 1990 to 2019.ASMR, age-standardized mortality rate; SDI, sociodemographic index

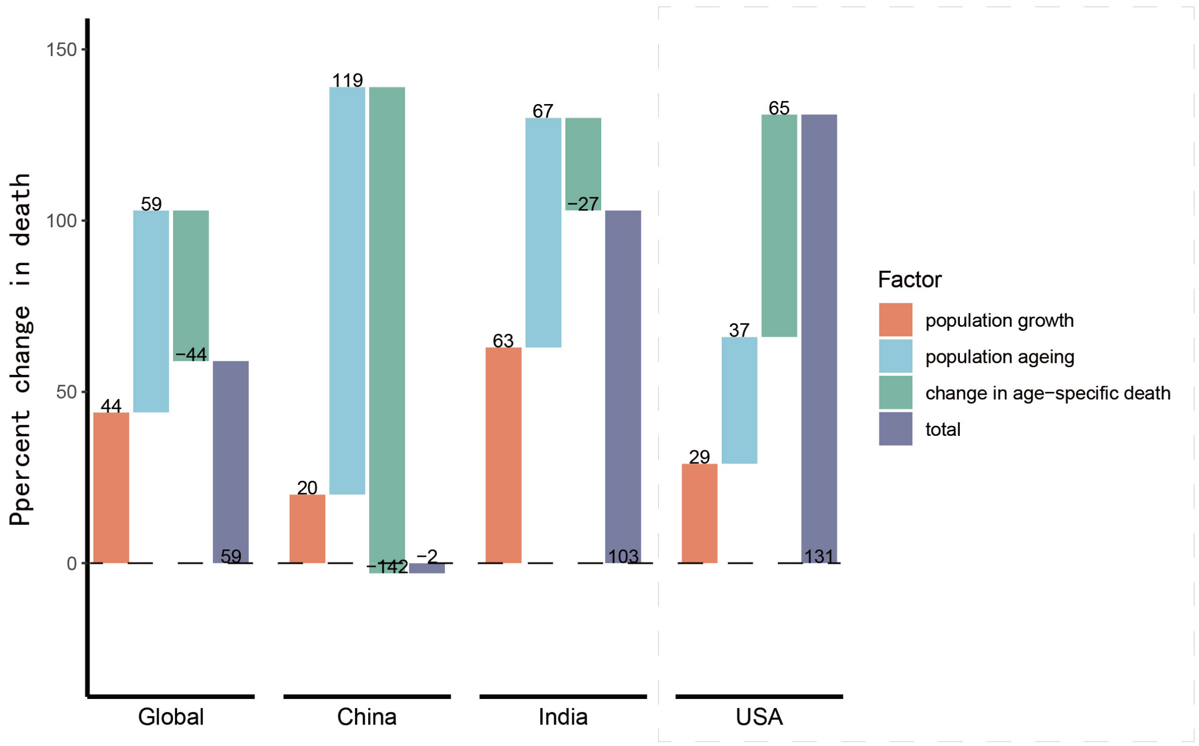


Figure S7. ASMR of HCV burden in different regions by SDI from 1990 to 2019.ASMR, age-standardized mortality rate; SDI, sociodemographic index
